# The Influence of Mechanical and Microstructural Characteristics on the Durability of a Femoral Implant Made of Different Alloys

**DOI:** 10.3390/jfb17060275

**Published:** 2026-06-02

**Authors:** Ivan Panfilov, Evgeniy Sadyrin, Andrey Nikolaev, Pavel Antipov, Andrey Vasiliev, Ilya Vilkovyskiy, Andrei Pantiulin, Oxana Ananova, Besarion Meskhi

**Affiliations:** 1 Department of Theoretical and Applied Mechanics, Agribusiness Faculty, Don State Technical University, 1 Gagarin Square, 344003 Rostov-on-Don, Russia; esadyrin@donstu.ru; 2 Scientific and Educational Center “Materials”, Don State Technical University, 1 Gagarin Square, 344003 Rostov-on-Don, Russia; andreynicolaev@eurosites.ru (A.N.); sly_fox_03@mail.ru (P.A.); andre.vasiliev@gmail.com (A.V.); 3 Department of Veterinary Medicine, RUDN University, 6 Miklukho-Maklaya St., 117198 Moscow, Russia; med-vet@bk.ru (I.V.); a.pantylin@gmail.com (A.P.); 4 Department of Marketing and Engineering Economics, Faculty of Innovative Business and Management, Don State Technical University, 344003 Rostov-on-Don, Russia; o_ananova@mail.ru; 5 Department of Life Safety and Environmental Protection, Faculty of Life Safety and Environmental Engineering, Don State Technical University, Gagarin, 1, 344003 Rostov-on-Don, Russia; spu-02@donstu.ru

**Keywords:** femoral implant, titanium alloy, finite element analysis, dynamic loading, stress concentrators, microtomography, nanoindentation, microstructure, chemical composition, reverse engineering

## Abstract

The long-term success of orthopedic implants is fundamentally dependent on the synergy between mechanical performance and biological integration. Thus, a comprehensive investigation of both mechanical characteristics and microstructural parameters is essential for the development of reliable implant systems in hip arthroplasty, both in human medicine and veterinary practice. The present study provides a detailed analysis of the mechanical properties, microstructure, and chemical composition of a Ti-6Al-4V-based femoral implant using nanoindentation, scanning electron and optical microscopy, and energy-dispersive X-ray spectroscopy. Then, using finite element analysis, the influence of Young’s modulus on the stress–strain state of the endoprosthesis was evaluated. Dynamic loading conditions were considered by analyzing an impact on a cantilever beam, simulating an animal’s jump onto a supporting limb. For reliable numerical simulation, the model geometry was constructed utilizing computed X-ray microtomography. The numerical simulations were performed for three material cases: reference Ti-6Al-4V, experimentally characterized Ti-6Al-4V (with properties determined by nanoindentation), and CoCrMo alloy, which is also widely used in endoprosthetic applications. The influence of the founded mechanical characteristics on the stress–strain state of the prostheses was assessed. In particular, the results indicate that under dynamic loading conditions, the load-bearing capacity of CoCrMo is lower by approximately 30% and 21% compared to the reference and experimentally characterized Ti-6Al-4V, respectively.

## 1. Introduction

Hip arthroplasty is one of the most commonly performed surgical procedures, aimed at relieving pain and restoring mobility in patients with severe joint pathologies (e.g., osteoarthritis) [1,2,3], post-traumatic conditions, and within the elderly population. According to World Health Organization data [4], the global population aged 60 years and older has increased significantly: in 2019, this demographic accounted for 13.2% of the total population, representing a 2.5-fold increase compared to 1980. The number of older adults is projected to reach nearly 2.1 billion by 2050 [5]. In 2010, an estimated 22 million women and 5.5 million men in the EU suffered from osteoporosis; consequently, 3.5 million new fragility fractures occurred, including 620,000 hip fractures [6]. According to data from the Royal College of Physicians in the UK (2007–2018) [7], 30-day mortality following hip fractures remains a concern, though rates have declined consistently in recent years. Reference [8] argues that total hip arthroplasty can be considered the ‘operation of the century’ for numerous reasons. Furthermore, [9] outlines promising development trends for implants based on a critical analysis of modern prosthetic designs, implantation techniques, and systemic postoperative complications.

Similarly to developments in human medicine, there is a growing demand for advanced orthopedic interventions in veterinary practice [10,11,12,13]. Companion animals, particularly dogs, frequently suffer from debilitating conditions such as hip dysplasia and osteoarthritis that severely impair mobility and quality of life. Although diagnostic screening and controlled breeding strategies have been implemented, hip dysplasia persists as a prevalent inherited musculoskeletal disorder in dogs [14,15]. According to major canine registries, including the American Kennel Club and the Fédération Cynologique Internationale, the overall prevalence of hip dysplasia in North America is approximately 15.6% [16], with a prevalence in predisposed breeds exceeding 60% [17]. This high clinical burden is reflected in market dynamics: the canine segment dominates the veterinary orthopedics market with an estimated share of 42.6% projected for 2025 [18], while the European veterinary orthopedics market alone was valued at USD 518.9 million in 2023, with a projected compound annual growth rate of 8.3% through 2030 [19].

Notably, the fundamental design architecture of hip prostheses exhibits significant convergence between human and veterinary applications, as both rely on similar biomechanical principles to restore joint function [20,21]. This structural similarity underscores the value of cross-species technological transfer. Moreover, in vivo assessment in animal models remains a critical step in validating the clinical efficacy, biocompatibility, and mechanical stability of orthopedic implants [22,23,24,25]. Therefore, rigorous evaluation within veterinary practice not only enhances animal care but also contributes to the broader understanding of prosthetic performance under physiological conditions.

Total hip arthroplasty predominantly employs four main bearing couple configurations: metal-on-polyethylene, metal-on-metal, ceramic-on-ceramic, and ceramic-on-polyethylene [26]. Novel hybrid options, including ceramic-on-metal, have also been developed to expand available choices [27,28]. Determining the optimal bearing involves weighing multiple criteria, such as cost-effectiveness, patient demographics, activity demands, and clinical considerations [29,30].

High-performance polymers, including polytetrafluoroethylene (PTFE), ultra-high molecular weight polyethylene (UHMWPE), and polyetheretherketone (PEEK) [31], have been extensively investigated for orthopedic applications. Katti, in a comprehensive review on the biomaterials in total joint replacement [32], remarks that during the early 1960s, total hip arthroplasty procedures involved articulating a stainless steel femoral component against a PTFE acetabular cup. However, due to excessive wear rates and mechanical deformation, PTFE was subsequently abandoned as a viable material for acetabular bearings. The primary tribological limitation of UHMWPE in joint arthroplasty is the generation of microscopic wear debris, which elicits an adverse inflammatory response in periprosthetic tissues and ultimately leads to aseptic loosening of prosthetic components [33]. Consequently, the clinical longevity of UHMWPE-bearing implants is typically restricted to 15–20 years [34]. Spectroscopy, microscopy, nanoindentation and calorimetry analyses were performed to provide information on the mechanism of wear acting in vivo in relation to wear rate per year, oxidation activities, and changes in mechanical properties of UHMWPE acetabular cup in total hip replacement by Shahemi et al. [35]. PEEK and its composites are polymers resistant to fatigue strain, radiologically transparent, and have mechanical properties suitable for a range of orthopedic applications [36,37]. While PEEK’s optimal mechanical and physical properties have promoted its adoption as an alternative to standard biomaterials, significant barriers persist. Specifically, the polymer’s bioinert and hydrophobic nature impedes protein adsorption and cellular adhesion required for effective osseointegration, potentially affecting long-term implant outcomes [38]. Therefore, further research is demanded to address these limitations.

Conventional metallic materials frequently employed in total hip arthroplasty include stainless steel, titanium alloys (e.g., Ti-6Al-4V [39]), and cobalt–chromium–molybdenum (CoCrMo) alloys. Owing to specific alloying additions and the formation of a protective chromium oxide (Cr_2_O_3_) passive layer, cobalt-based alloys demonstrate exceptional corrosion resistance and mechanical strength in chloride-containing environments, distinguishing them from most other metallic biomaterials [40]. Supporting this, Manthe et al. [41] utilized mechanical, electrochemical, and surface characterization data to demonstrate the superior resistance of CoCrMo hip implants to fretting-corrosion damage. Regarding titanium systems, Fellah et al. [42] investigated the friction and wear behavior of the high-strength Ti-6Al-7Nb alloy used in femoral stems, comparing it with conventional Ti-6Al-4V. Finite element analysis by Şensoy [43], utilizing CES Selector 2014 software, demonstrated that annealed austenitic stainless steel effectively minimizes micromotion at the implant–cement interface, supporting conditions favorable for osseointegration. Porous tantalum represents an alternative metallic biomaterial for total joint arthroplasty components, offering several unique properties. Its high volumetric porosity (70–80%), low modulus of elasticity (~3 GPa), and high frictional characteristics promote biologic fixation [44]. Experimental data [45] indicate that porous tantalum is effective for biologic fixation in canine models and may provide a suitable alternative to other porous materials used in acetabular cup design.

Ceramic bearing surfaces are increasingly utilized in total hip arthroplasty, with favorable mid- to long-term outcomes reported owing to their outstanding tribological properties [46,47,48]. Despite advancements in modern ceramic manufacturing, fracture events still occur and must be minimized to ensure a viable clinical option with acceptable risk profiles [49,50,51]. Among common ceramics for hip endoprostheses, alumina (Al_2_O_3_) deserves mention [52]; however, recent clinical reports have raised concerns regarding audible squeaking in certain cases [53]. Zirconia ceramics offer several advantages over other ceramic materials due to transformation-toughening mechanisms within their microstructure, which impart superior mechanical properties to the components [54]. Notably, De Aza et al. [55] demonstrated that for orthopedic applications, alumina–zirconia composites exhibit higher reliability than monolithic ceramics, attributed to enhanced fracture toughness.

The long-term success of orthopedic implants is fundamentally dependent on the synergy between mechanical performance and biological integration. Optimal osseointegration requires not only biocompatible surfaces but also mechanical properties that closely approximate those of native bone tissue. When implant stiffness significantly exceeds that of the surrounding bone, stress shielding may occur, potentially leading to periprosthetic bone loss and aseptic loosening. In addition, microstructural features play a pivotal role in cellular response and the stability of the bone–implant interface. Therefore, a thorough examination of mechanical properties and microstructural details is crucial for creating dependable implant systems. In this context, the present study provides a detailed analysis of the mechanical properties, microstructure, and chemical composition of a Ti-6Al-4V-based femoral implant designed for veterinary applications. Additionally, the effect of Young’s modulus on the stress–strain condition of the endoprosthesis is examined. To account for dynamic loading conditions, an impact on a cantilever beam is analyzed to simulate a jump onto a supporting limb.

## 2. Materials and Methods

**Figure 1:**
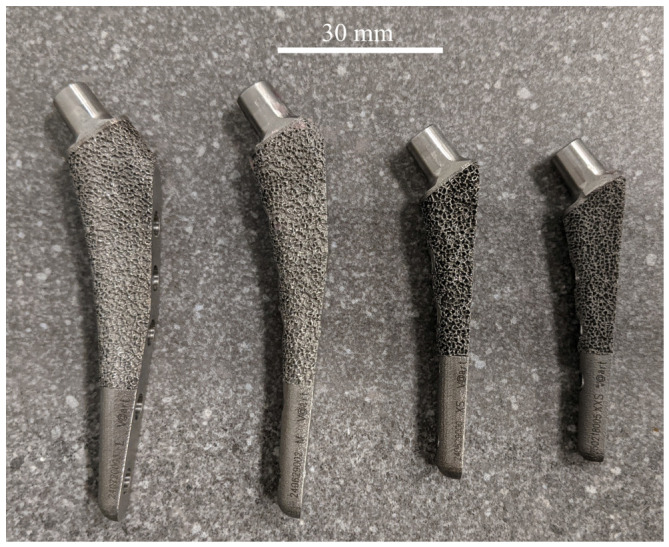
The set of the V@rt canine endoprostheses.

**Figure 2:**
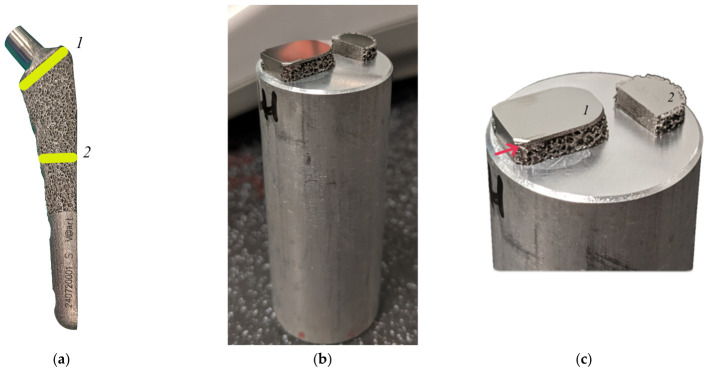
The investigated endoprosthesis: (**a**) general view of the sample; (**b**) sections prepared for nanoindentation view 1; (**c**) sections prepared for nanoindentation view 2, SEM, and EDX analysis. Yellow lines indicate the approximate positions of the cut sections: (1) near the neck (proximal end of the stem) and (2) at the approximate center of the stem. A narrow unroughened rim at the extreme proximal end, adjacent to the neck, is indicated by the red arrow.

Figure 1 displays the V@rt canine endoprostheses [56]. The complex of mechanical characteristics was collected from the S-size implant (Figure 2a), specifically examining the stem component, which is inserted into the femoral canal. The prosthesis size is determined by the dog’s weight and anatomical dimensions. During the procedure, a neck component is attached to the stem; this part subsequently articulates with the corresponding pelvic structures (cup and head). The sample was mounted vertically on the holder with dental wax to minimize the offset from the rotation axis (Figure 2b).

While computational modeling of implants often utilizes literature-based material data, actual physical and mechanical properties may differ from nominal values [57,58]. Therefore, this study prioritizes the experimental characterization of properties at different sites on a manufactured device to enhance modeling accuracy. Thus, we conduct a more detailed analysis of the mechanical behavior of the implant. Additionally, the as-built geometry is employed instead of the nominal STL file, addressing known geometric deviations inherent to additive manufacturing processes [59,60].

To capture the digital geometry of both the S- and XS-type implants, a Xradia Versa 520 micro-CT system (Carl Zeiss X-ray Microscopy, Inc., Pleasanton, CA, USA) was utilized. The scanning parameters were set as follows for both samples: X-ray tube voltage of 110 kV, power of 9 W, and no tube filtration. Pixel sizes of 56.0 and 40.01 μm, as well as exposure times of 1.0 and 1.2 s, were applied for the S- and XS-type samples respectively. To enhance the resolution of the final tomogram, scanning was conducted in vertical stitching mode with 0.4× optical magnification. The samples underwent two and three separate 360° scans (for upper and lower segments) for both the S- and XS-type implants, respectively, acquiring 1601 X-ray projections per segment. The X-ray source filters were selected based on the observed transmittance values according to the recommendations of the Xradia Versa 520 User’s Guide A003030 Rev. B. These segments were subsequently merged using Vertical Stitcher software (v.11.0.4241.15713).

For each scan, the sample was positioned as close as possible to the X-ray source (source-to-axis distance: 104.10 and 104.11 mm; detector-to-axis distance: 24.0 mm and 75.17 for the S- and XS-type implants, respectively). The 2048 × 2048 pixel CCD camera was cooled to −59 °C. Data acquisition employed a camera binning factor of 2, yielding projection images of 1024 × 1024 pixels. Compensatory motions were applied to correct for sample drift during scanning.

**Figure A1:**
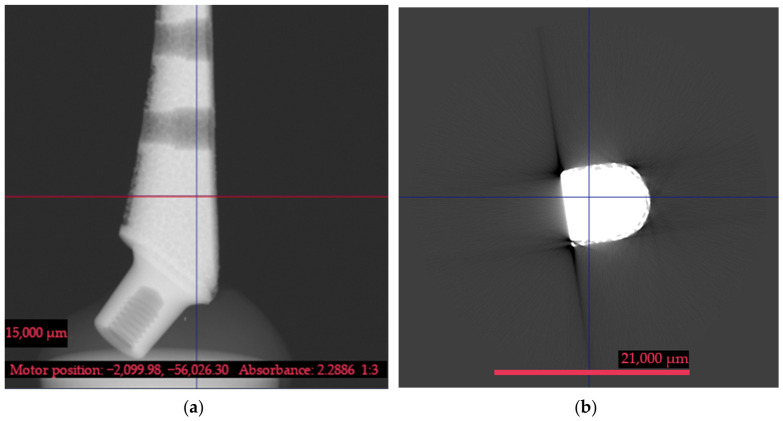
The typical projections used for reconstruction process of the XS-type implant, upper segment: (**a**) selection of the horizontal projection to perform the required reconstruction; (**b**) the reconstructed projection.

Reconstruction of the projection data into virtual slices with TXM resolution was performed using XRMReconstructor software (version 12.0). Center shift values were defined (18.2 and 18.6; 18.47, 18.636 and 18.407 for the respective tomograms for the S- and XS-type implants,, respectively; see Figure A1, Appendix A for reference). Post-processing included the application of a Gaussian blur filter (0.7) and a beam spectrum hardening shift (0.1 and 0.05 for the S- and XS-type implants respectively). A warm-up scan was conducted prior to each data acquisition. For three-dimensional visualization, rendering was performed using the Phong shading model [61]. Finally, the segmented volumes were converted into STL models for further FEM analysis, ensuring that the reconstructed geometry accurately represents the physical sample using Vesalius 3.1.1 software. The segmentation of the implant geometry and its internal structure was conducted using a global manual thresholding, based on the grayscale histogram analysis.

Following the micro-CT analysis, wax residues were removed from the sample, and it was sectioned using a precision saw (Isomet 4000, Buehler, Lake Bluff, IL, USA) equipped with an Al_2_O_3_ abrasive cutting disk (Met-Disc-T, MetCata GmbH, Kaufering, Germany). Two parallel cross-sections were obtained: one in the vicinity of the neck (the proximal end of the stem) and the other approximately at the center of the stem. Parallelism between the sections was ensured using the integrated micrometer adjustment system of the saw.

Initial grinding was performed on a MetaServ 250 grinder–polisher (Buehler) using Al_2_O_3_ disks (P220 and P1200 grit) under continuous water cooling at 250–300 rpm for 3 min each, with loads of 5–10 N and 10 N, respectively. Subsequent polishing involved multiple stages using 15 μm, 9 μm, and 3 μm diamond suspensions (15–20 N load) with Greenlube lubricant (Allied High Tech Products Inc., Cerritos, CA, USA) to ensure surface smoothness without altering the native microstructure (Figure 2c).

The mechanical properties of the endoprosthesis neck and stem were investigated via nanoindentation using a Nanotest 600 Platform 3 system (Micro Materials Ltd., Wrexham, UK) equipped with a Berkovich diamond indenter. The indents were performed under a peak load of 50 mN, arranged in a pattern of six columns with four indents per column. The inter-column spacing was set as follows: 100 μm between columns 1 and 2; 200 μm between 2 and 3; 300 μm between 3 and 4; 400 μm between 4 and 5; and 500 μm between 5 and 6. The spacing between the first series and sample edge was 30 μm. This arrangement allowed for the assessment of property variations as a function of depth from the surface. This indentation pattern was applied identically to both samples. Additionally, optical micrographs of nanoindentation impressions were made using the microscope positionally synchronized with the force cell of the platform. The loading rate was 1.8 mN/s. A dwell time of 10 s was applied at peak load. Drift correction was taken into account during the experiment analysis. Drift correction data was collected during both loading and unloading for each indentation into the material. Mechanical properties were calculated using the Oliver–Pharr method [62]. A Poisson’s ratio of 0.34 [63] was assumed for the alloy to calculate Young’s modulus.

The surfaces of the sections were visualized using a Crossbeam 340 scanning electron microscope (SEM, Carl Zeiss Microscopy GmbH, Oberkochen, Germany) using an Everhart–Thornley secondary electron detector [64] at an accelerating voltage of 3–5 kV and an aperture size of 30/60 μm. AZtec 6.3 Energy Advanced with a nitrogen-free detector X-Max 80 (Oxford Instruments Ltd., Wycombe, UK) was used for energy-dispersive X-ray spectroscopy (EDS).

## 3. Results and Discussion

### 3.1. Experimental Studies

**Figure 3:**
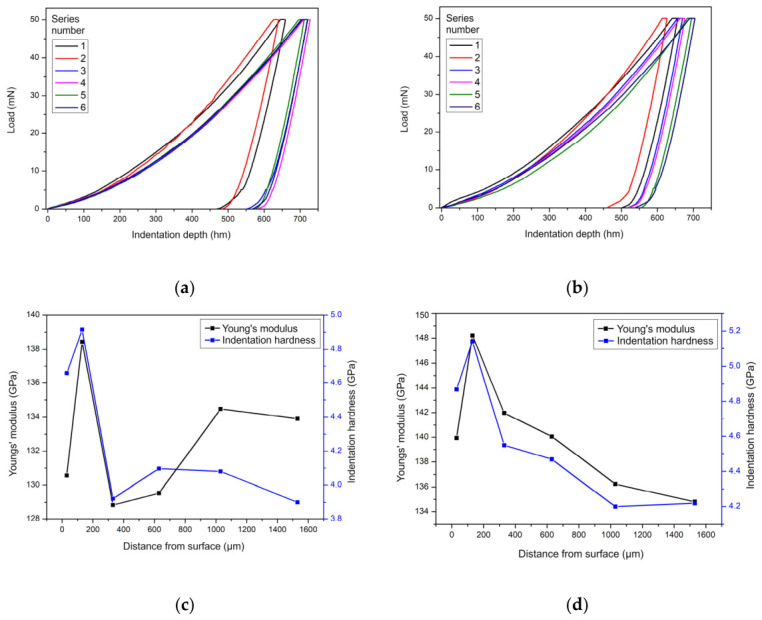
Nanoindentation results for peak load of 50 mN: (**a**) load–displacement curves for the section near the neck (proximal end of the stem); (**b**) load–displacement curves for the section at the stem center; curves 1, 2, 3, 4, 5 and 6 correspond to representative indentation results obtained in an area 30 μm, 130 μm, 330 μm, 630 μm, 1030 μm, and 1530 μm from the sample edge; (**c**) mechanical properties (hardness and elastic modulus) as a function of depth from the surface for the neck-proximal section; (**d**) mechanical properties as a function of depth from the surface for the central stem section.

The nanoindentation results for sections taken near the neck (proximal end) and at the center of the stem are presented in Figure 3. Although the individual load–displacement curves exhibit similar behavior for both regions, several notable trends emerge. Both the neck and stem regions show a comparable pattern: Young’s modulus and hardness increase at a depth of approximately 130 μm from the implant edge surface, following a reduction in properties observed at 30 μm from the edge.

This near-edge reduction is attributed to boundary effects, where the proximity of the sample edge reduces the material resistance during indentation, resulting in lower apparent mechanical properties. Beyond this near-edge region, mechanical properties gradually decrease with increasing distance from the outer surface of the endoprosthesis toward the bulk material. However, this trend is non-monotonic and more pronounced in the central stem section. Quantitatively, Young’s modulus and hardness in the neck region decrease by 9% and 20%, respectively, relative to near-surface values, while in the central stem region, the corresponding reductions are 9% and 18%. Furthermore, Figure 3c and Figure 3d indicate that, on average, the central stem region exhibits Young’s modulus and hardness values approximately 6% and 7.4% higher, respectively, than those measured in the neck region. At lower peak loads of 10 mN, indentation measurements yielded higher Young’s modulus values, corresponding to the near-surface material (146.28–154.42 GPa), while the indentation hardness ranged from 5.26 to 5.52 GPa.

**Figure 4:**
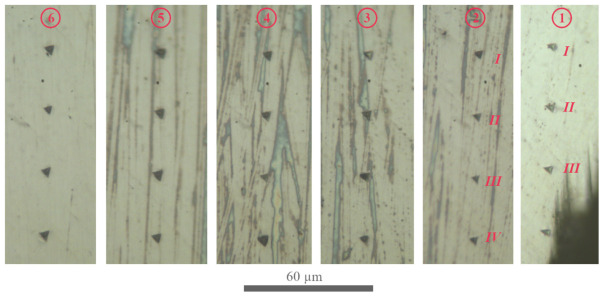
Optical micrographs of nanoindentation impressions for the section near the neck (proximal end of the stem). The outermost indentations (1) are shown in the rightmost panel; indents progress from the sample edge (**right**, **panel 1**) toward the inner portion (**left**, from **panel 1** to **panel 6**), with the number of series designated in the upper portion of each panel corresponding to those from Figure 2a. The numbers I–IV designate the SEM-observed indents.

**Figure 5:**
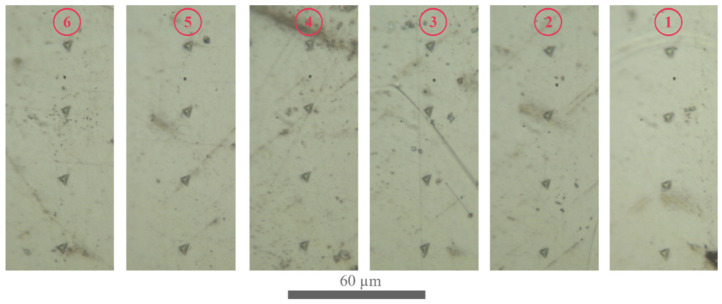
Optical micrographs of nanoindentation impressions for the section at the stem center. The outermost indentations are shown in the rightmost panel (1); indents progress from the sample edge (**right**, **panel 1**) toward the inner portion (**left**, from **panel 1** to **panel 6**), with the number of series designated in the upper portion of each panel corresponding to those from Figure 2b.

The optical observation of the nanoindentation imprints for the section near the neck (proximal end of the stem) are shown in Figure 4, and for the section at the stem center shown in Figure 5.

**Figure 6:**
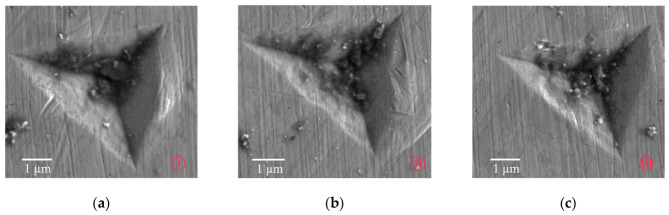
SEM images of the nanoindentation impressions, section near the neck (proximal end of the stem), first line near the sample edge: (**a**) uppermost impression (I); (**b**) second impression (II); (**c**) third impression (III).

**Figure 7:**
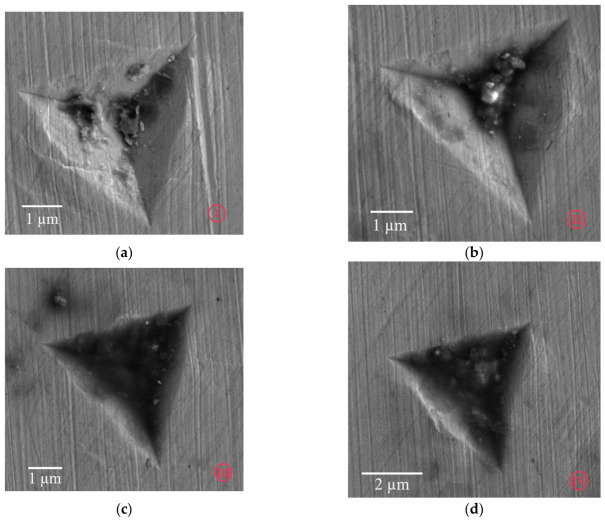
SEM images of the nanoindentation impressions, section near the neck (proximal end of the stem), second line near the sample edge: (**a**) uppermost impression (I); (**b**) second impression (II); (**c**) third impression (III); (**d**) lower impression (IV).

SEM imaging was performed on residual nanoindents in the neck (proximal end of the stem) section. The three indentations closest to the sample edge (Figure 4, panel 1) are shown in Figure 6, and four indentations from the subsequent row (Figure 4, panel 2) are presented in Figure 7 with the microgeometrical features of the surface of the impressions [65,66].

**Figure 8:**
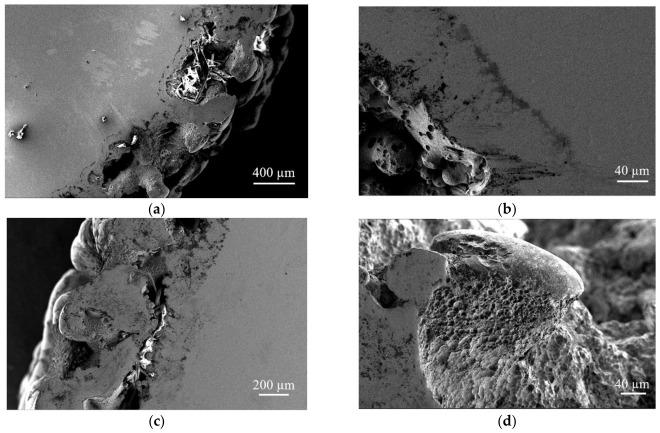
SEM images of the roughened implant texture: (**a**) general view of the surface fabricated to promote osseointegration; (**b**) cross-section showing the subsurface/inner structure of the material; (**c**) magnified view of specific microgeometric surface features; (**d**) a single characteristic surface element.

Approximately two-thirds of the stem surface, primarily in the region adjacent to the neck (excluding a narrow rim at the extreme proximal end; see Figure 2c), exhibits a roughened texture designed to facilitate bone ingrowth and osseointegration. Representative SEM micrographs of this surface morphology are presented in Figure 8.

**Figure 9:**
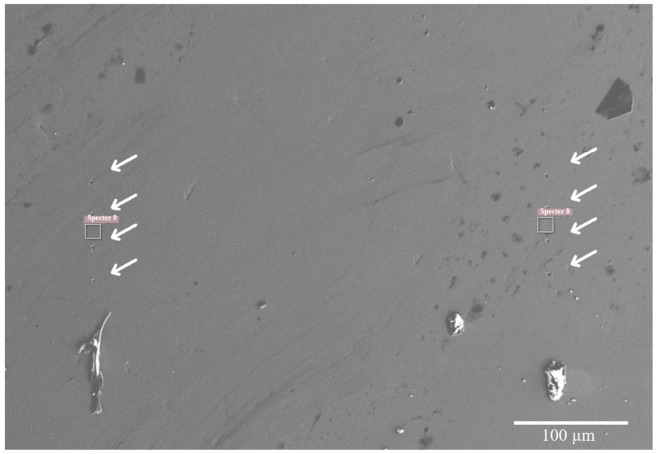
EDS measurements. The arrows indicate the indentation impressions; the white rectangle marks the area where data were collected.

**Figure 10:**
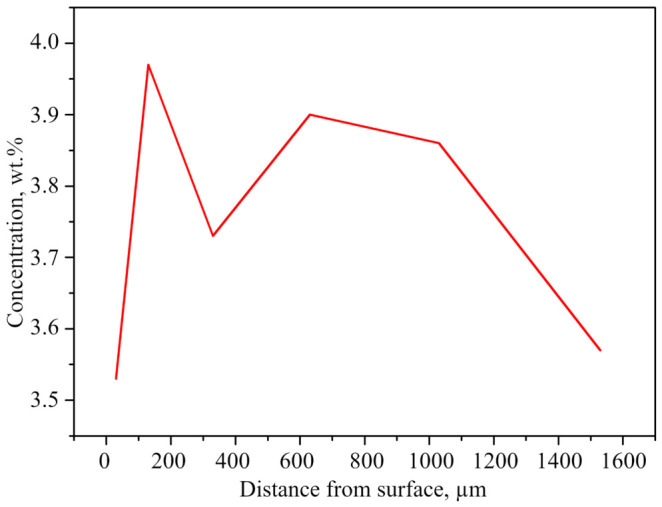
Vanadium concentration wt. % as a function of depth from the surface for the neck-proximal section.

Given that typical failures of the investigated implant occur near the neck region [11], further compositional analysis using EDS was conducted to elucidate the causes of fatigue failure in these endoprostheses. Data collection was performed as follows: measurements were acquired within a rectangular area located between the second and third indents in each series (Figure 9); where necessary, additional measurements were taken between other adjacent indents. The resulting chemical composition data are summarized in Table 1. Notably, the Al and V contents are in good agreement with the state standard [67]. The vanadium concentration profile (Figure 10) exhibits a spatial distribution trend similar to that of indentation hardness in the neck region (Figure 3c), suggesting a correlation between local chemistry and mechanical properties.

**Figure 11:**
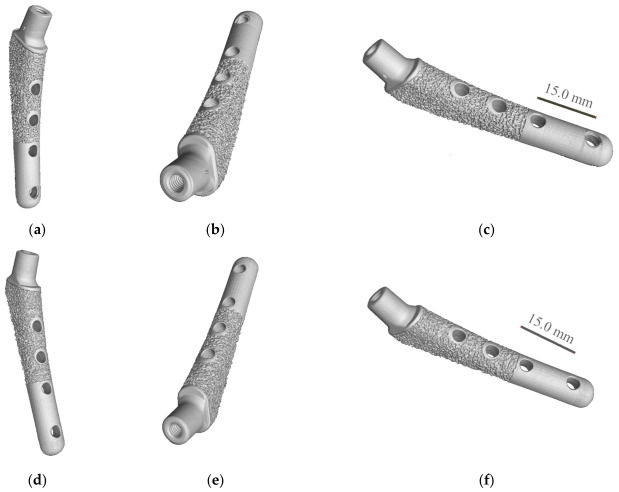
Micro-CT 3D reconstructions of the samples at different viewing angles: (**a**–**c**) S-size type; (**d**–**f**) XS-size type.

Non-destructive evaluation of implant quality prior to clinical application is of critical importance, particularly due to the potential presence of internal defects and structural artifacts. Among the available techniques, computed micro-CT has emerged as an effective tool for the analysis of defects and porosity [68,69]. This method is based on the acquisition of multiple projection images of a specimen during rotation, which are subsequently reconstructed into a three-dimensional volume composed of voxels. The resulting dataset, either in full or in part, can then be further processed and analyzed within a digital environment [70,71]. A key advantage of micro-CT over conventional computed tomography lies in its superior spatial resolution. While the voxel size in conventional computed tomography is typically on the order of 1 mm^3^, micro-CT enables resolutions below 10 µm^3^ [72,73]. This high resolution allows for an accurate representation of the actual geometry, including manufacturing-induced features, and facilitates the identification of microcracks, pores, and inclusions. Previous studies have shown that components produced using additive manufacturing techniques may deviate from their original computer-aided design models at the microstructural level [74,75]. Therefore, accurate characterization of the as-built geometry is essential for reliable numerical modeling of the stress–strain behavior of implants [76], thus enabling a reverse-engineering approach. Figure 11 presents volumetric renderings of the sample obtained using micro-CT, illustrating the surface structure of S-size and XS-size implants and enabling detailed assessment of potential defects.

It is worth noting that the average Young’s modulus obtained from the nanoindentation tests (E = 137.40 GPa) is slightly higher than the values reported in the literature. For instance, a review [77] reports a value of 110 GPa for a water-quenched ELI alloy after solution treatment. Similarly, Trofimov et al. [78] reported values ranging from 89.4 to 117.4 GPa depending on the grain size, using both tensile testing and nanoindentation, while the reference book [79] specifies a range of 100–110 GPa. Comparable values were provided in [80]. In [81] the observed values of 112.8 GPa for wrought materials and 109.9–118.8 for selective laser melting technology were reported.

Despite the consistent trends observed, it is important to acknowledge the limitations associated with the application of the Oliver–Pharr method in this study. The classical Oliver–Pharr model assumes a monolithic, homogeneous half-space, which may not fully capture the complex behavior of an alloy with structural heterogeneity or gradient properties. In our case, the potential uncertainty introduced by surface roughness was minimized through rigorous metallographic polishing and systematic line indentation on cross-sections. By treating the material as locally homogeneous at each depth increment, we obtained a reliable mechanical profile that can be considered a first approximation for the deeper study. However, this approach primarily provides an effective response of the material. Future research incorporating advanced modeling [82,83,84,85,86,87] would be beneficial to further refine these results and account for the discrete microstructural influences on the indentation response.

### 3.2. Numerical Study of the Influence of Young’s Modulus on the Stress–Strain State of an Endoprosthesis

**Figure 12:**
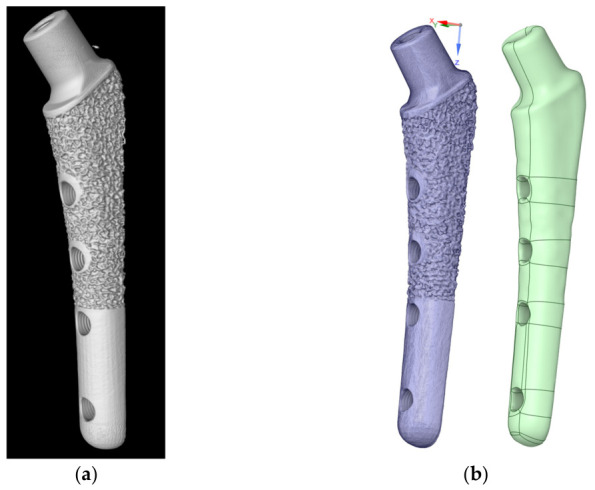
V@RT XS implants. (**a**) Scan geometry in STL format; (**b**) gray—original geometry after scanning in STL format, green—3D geometry in STP format prepared for calculation.

The influence of Young’s modulus on the stress–strain state of the endoprosthesis was investigated using numerical simulation. A test problem was formulated for a size XS prosthesis (Figure 12) and analyzed in the Ansys Mechanical 2022R1 finite element software for materials with different Young’s moduli.

**Figure 13:**
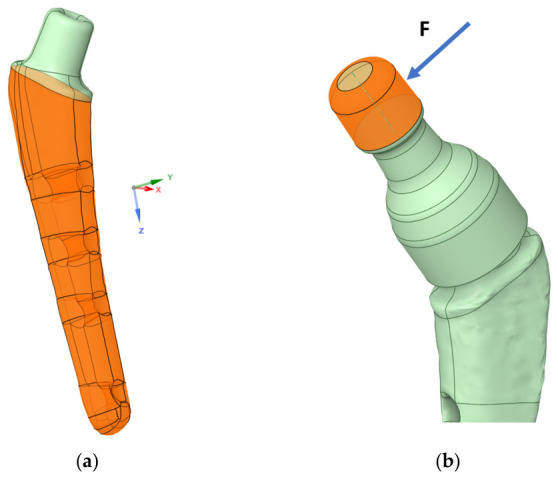
Fixation and load on the implant: (**a**) blue—fixation; (**b**) load on the neck.

For the simulation, the rough surface intended for bone tissue ingrowth was removed from the prosthesis (Figure 12), as it is expected to have only a local effect on the overall stress–strain state. To simplify the problem formulation, fixation was assumed to be uniform over the surface of the lower part of the prosthesis. Accordingly, the lower part of the prosthesis was modeled as rigidly fixed (Figure 13a), corresponding to continuous adhesion of the bone tissue. The influence of fixation conditions, including the effects of bone tissue and non-uniform fixation due to tissue ingrowth, has been discussed in [11,88,89,90].

A static load is applied to the upper part of the neck along an axis perpendicular to the longitudinal axis of the neck (Figure 13b). The test load is 294.3 N. Contact with a friction coefficient of 0.5 is established between the surfaces of the stem and the inner surface of the neck.

**Figure 14:**
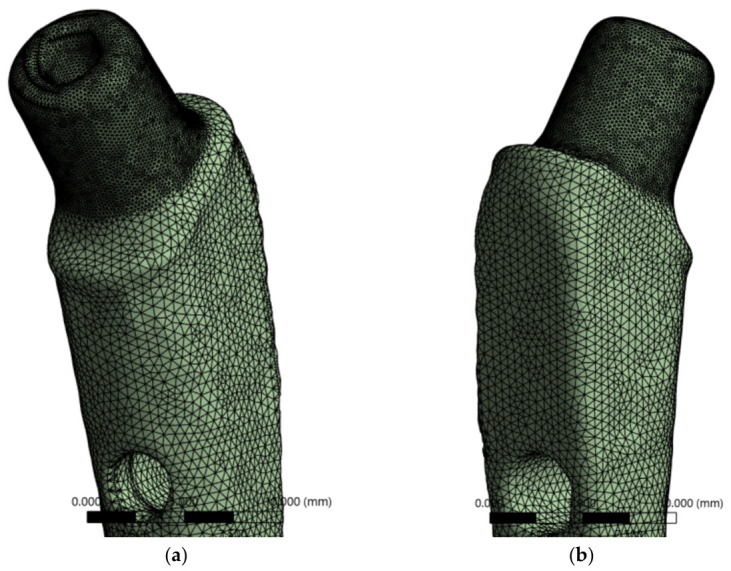
Finite element mesh: (**a**) general mesh; (**b**) mesh refinement in the neck area.

Figure 14 shows a finite element mesh of tetragonal elements with intermediate nodes, consisting of 327,779 nodes and 215,538 elements. The smallest mesh size is 0.2 mm, and the largest mesh size is 0.5 mm. Calibration tests were performed using the cell size. With a minimum mesh size of 0.1 mm and a maximum mesh size of 0.3 mm (1,192,242 nodes and 796,760 elements, respectively), the difference between the results for the mesh with the parameters specified above was less than 0.3%.

**Figure 15:**
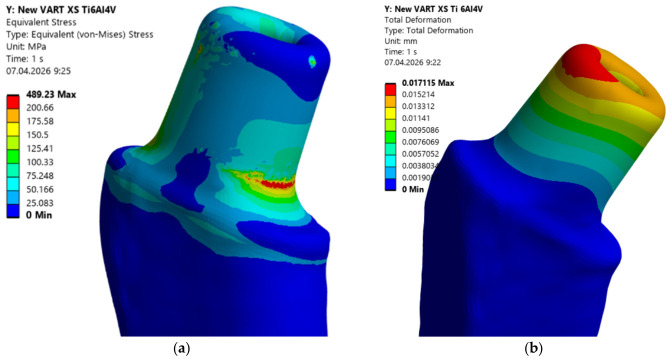
Stress–strain state for a prosthesis made of reference alloy Ti6Al4V, E = 117 GPa: (**a**) von Mises stress, (**b**) general deformations.

**Figure 16:**
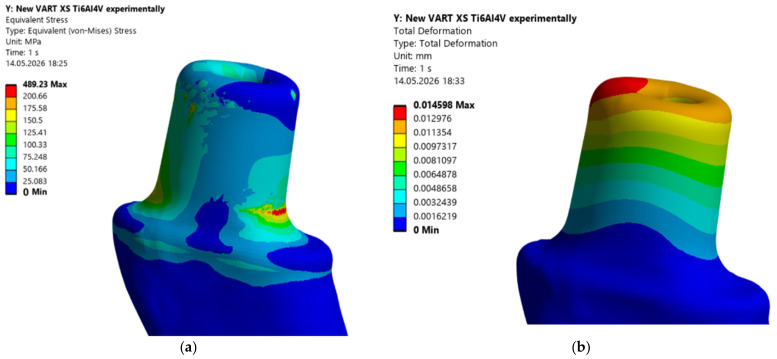
Stress–strain state for a prosthesis made of Ti6Al4V alloy, the characteristics of which were obtained experimentally, E = 137.4 GPa: (**a**) von Mises stress, (**b**) total deformations.

**Figure 17:**
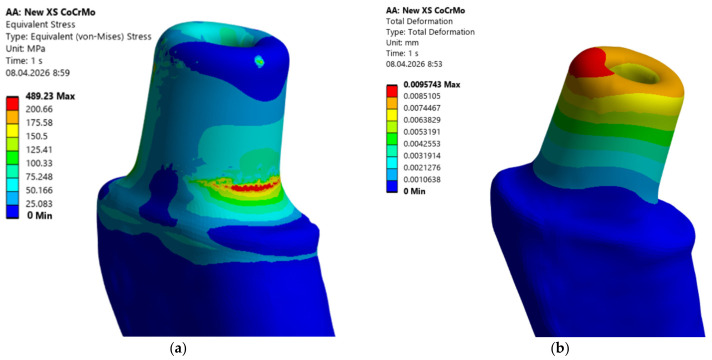
Stress–strain state for a prosthesis made of reference alloy CoCrM, E = 222 GPa: (**a**) von Mises stress, (**b**) general deformations.

Figure 15, Figure 16 and Figure 17 below show the von Mises stress and total strain results for the reference values of Young’s modulus at 117 GPa and the average Young’s modulus at 137.4 GPa, obtained by nanoindentation of the Ti6Al4V alloy. Also included for comparison are results for the CoCrMo alloy [11,91], which is also used in practice to manufacture endoprostheses. The Young’s modulus for this alloy is 222 GPa [92], and the yield strength and tensile strength are comparable to those of Ti6Al4V alloys. The alloy characteristics are presented in Table 2. The results of maximum stresses and strains are shown in Table 3.

The numerical analysis is performed using elastic, geometrically linear materials Ti6Al4V and CoCrMo, respectively. Solver Type is Direct. The Newton–Raphson method with a Force Convergence of 0.5% is used for calculations.

Maximum von Mises stresses $\sigma_{M}$ occur at the lower part of the stem at the neck edge. This fact is confirmed by failures of prostheses at this point of stress concentration, which are encountered in medical practice [11].

The smallest deformations were expectedly obtained for the prosthesis made of the alloy with the highest Young’s modulus—the CoCrMo alloy. The ratio of the maximum displacements for these alloys (Table 3) is directly proportional to the ratio of Young’s moduli, which is due to the linear theory of elasticity of small deformations of the equations used for the numerical finite element method [93,94,95].

**Figure 18:**
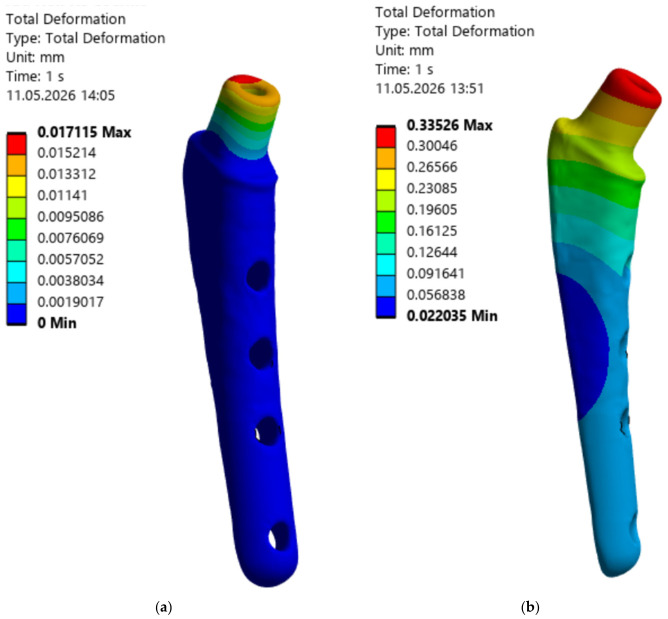
Deformations for prosthesis, E = 117 GPa: (**a**) rigid fixation, (**b**) elastic fixation.

**Figure 19:**
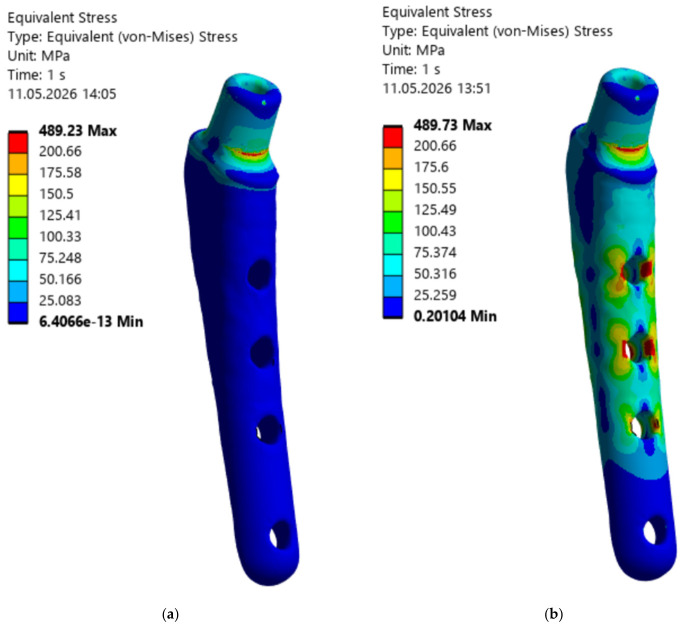
Stress for prosthesis, E = 117 GPa: (**a**) rigid fixation, (**b**) elastic fixation.

To assess the influence of boundary conditions on the bone, additional calculations were performed for an elastic support with a modulus of 18 GPa, which corresponds to the stiffness of the femur. Figure 18 and Figure 19 below show the stresses and strains for a rigidly clamped prosthesis and an elastically clamped one for Ti6Al4V, E = 117 GPa.

It should be noted that for rigid support, the stress concentrator is located in the upper part of the stem, while for elastic support, the stress concentrators are shifted to the lower part above the holes for the bicortical screws. This fact is quite interesting and requires separate research: in particular, the influence of the degree of biointegration (fusion) of the prosthesis with the bone on the strength properties of the prosthesis and the bone itself.

To account for dynamic loading conditions, an impact on a cantilever beam subjected to a force *F* from a height *h* is considered, representing a jump onto a supporting limb with a corresponding mass. For simplicity, the damping effects of biological tissues are neglected. The dynamic coefficient *K* for increasing the static load is equal to [96]:

(1) $$K=1+\sqrt{1+\frac{2\cdot h}{\delta_{st}}},$$

where $\delta_{st}$ is the displacement due to a static load of 0.0171 mm, 0.0146 mm, and 0.0096 mm, respectively. For axial impact, Equation (1) takes an analogous form, with the direction of the applied load *F* appropriately modified.

**Figure 20:**
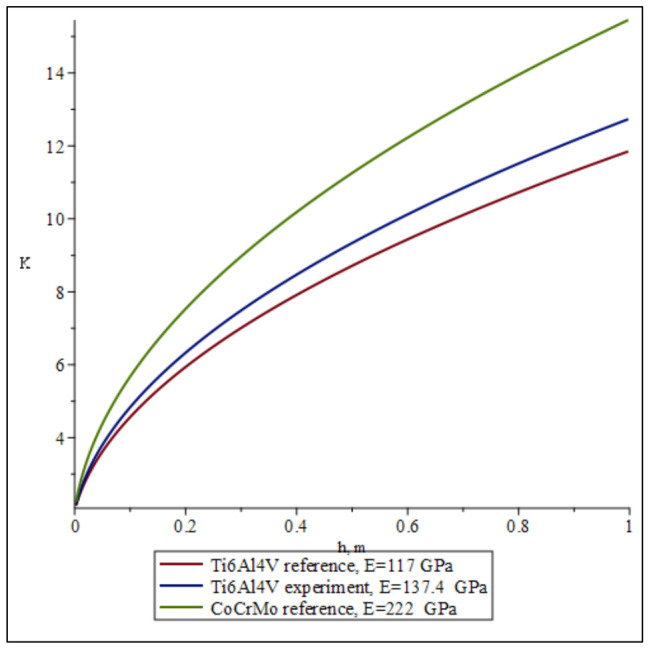
Values of dynamic coefficients for the range h from 0 to 1 m for different alloys.

Figure 20 shows the values of the dynamic coefficients for the range *h* from 0 to 1 m.

Assuming that the yield strength and tensile strength of Ti6Al4V and CoCrMo alloys are comparable according to Table 2, then under dynamic loads for a more “rigid” material, the stress values and, consequently, the load-bearing capacity according to the criterion of inadmissibility of plastic deformations (2)–(3) will be less than for “softer” alloys.

(2) $$\sigma_{M}\leq \;K\cdot \sigma_{T},$$

(3) $$\sigma_{M}=\sqrt{\frac{{\left(\sigma_{xx}-\sigma_{yy}\right)}^{2}+{\left(\sigma_{yy}-\sigma_{zz}\right)}^{2}+{\left(\sigma_{zz}-\sigma_{xx}\right)}^{2}+6\left(\tau_{xy}^{2}+\tau_{yz}^{2}+\tau_{zx}^{2}\right)}{2}},$$

where $\sigma_{M}$ is the maximum von Mises stress, $\sigma_{ij}$ is the stress tensor components, and $\sigma_{T}$ is the yield strength from Table 2.

Thus, the dynamic coefficient for CoCrMo is 30% and 21% greater than the dynamic coefficient of reference Ti6Al4V and experimental Ti6Al4V, respectively, in the specified *h* ranges. In other words, the load-bearing capacity under dynamic loads for CoCrMo is 30% and 21% less than the load-bearing capacity of reference Ti6Al4V and experimental Ti6Al4V, respectively, in the specified *h* ranges. This factor can serve as a significant argument when choosing alloys for the manufacturing of endoprostheses. It should be noted that higher damping coefficients in a real skeletal system will reduce the dynamic coefficients, but the qualitative assessment will be preserved.

## 4. Conclusions

This study presents a comprehensive mechanical and microstructural characterization of a Ti-6Al-4V femoral implant, combining experimental techniques and numerical modeling. The obtained results demonstrate that local mechanical properties measured by nanoindentation reveal variations associated with near-surface regions.

Finite element analysis showed that the stress–strain state of the endoprosthesis is sensitive to the value of Young’s modulus, highlighting the importance of using experimentally determined material properties for accurate simulations. For fixed parameters, the deformation δ_st_ depends linearly on Young’s modulus, and the dynamic coefficient is described by Formula (1) from this displacement.

The incorporation of micro-CT-based geometry significantly improved the reliability of the numerical model by accounting for manufacturing-induced deviations from the nominal design.

Dynamic loading conditions, simulated through an impact scenario, revealed notable differences in load-bearing capacity between the investigated materials. In particular, the CoCrMo alloy exhibited a reduction in load-bearing capacity of approximately 30% and 21% compared to the reference and experimentally characterized Ti-6Al-4V, respectively.

Despite the comprehensive nature of the present study, several limitations should be acknowledged. The mechanical characterization performed by nanoindentation was based on conventional analysis approaches, which may not fully capture the influence of surface roughness, heterogeneity, and complex stress states inherent to additively manufactured materials. In future work, there are plans to implement more advanced methodologies for determining mechanical properties from nanoindentation data, particularly those based on rigorous solutions of contact mechanics problems. These approaches are expected to provide improved accuracy in evaluating elastic and plastic properties, especially in near-surface regions. Furthermore, the incorporation of more realistic boundary conditions, including the effects of bone tissue interaction and damping under dynamic loading, as well as validation against experimental mechanical testing will be considered to enhance the reliability of the numerical models.

Overall, the results emphasize the critical role of combined microstructural characterization and advanced numerical modeling in the design and assessment of orthopedic implants. The proposed approach can be effectively applied to improve the reliability and performance of endoprosthetic systems in both human and veterinary applications.

Future studies also plan to consider “more complex” theoretical models that explicitly take into account bone remodeling and damage evolution, which may contribute to increasing the predictive ability of the model, especially under physiological loading conditions [97,98].

## Figures and Tables

**Table 1:** Chemical composition of the sample, section near the neck (proximal end of the stem), concentration weight percent, wt. %.

Specter Number	Al, wt. %	Ti, wt. %	V, wt. %
Specter 1	5.91	90.56	3.53
Specter 2	5.54	90.48	3.97
Specter 3	5.61	90.66	3.73
Specter 4	5.48	90.15	4.37
Specter 5	5.52	90.62	3.86
Specter 6	5.84	90.59	3.57
Specter 7	5.67	90.78	3.55
Specter 8	5.48	90.72	3.80

**Table 2:** Characteristics of alloys for endoprostheses.

Alloy	Young’s Modulus, GPa	Yield Strength, MPa	Tensile Strength, MPa
Ti6Al4V reference	117	790	860
Ti6Al4V experiment	137.4	-	-
CoCrMo reference	222	750	816

**Table 3:** Results of maximum stresses and strains for different alloys.

Alloy	Maximum von Mises Stress, MPa	Maximum Total Deformations, mm
Ti6Al4V reference	489.2	0.0171
Ti6Al4V experiment	489.2	0.0146
CoCrMo reference	489.2	0.0096

## Data Availability

The original contributions presented in the study are included in the article; further inquiries can be directed to the corresponding authors.

## Abbreviations

The following abbreviations are used in this manuscript:

micro-CT	Computed X-ray microtomography
SEM	Scanning electron microscopy
EDS	Energy-dispersive X-ray spectroscopy
PTFE	Polytetrafluoroethylene
UHMWPE	Ultra-high molecular weight polyethylene
PEEK	Polyetheretherketone

## References

[1] Ishak-Samrin M., Naina-Mohamed I., Zulfarina M.S., Abdul Wahid S.F., Mohd Don A.F., Mohamad N., Ramlan M.K.R., Badrul A.H.M.Y. (2025). Treatment of Knee Osteoarthritis and Chondral Injury with Umbilical Cord/Wharton’s Jelly-Derived Mesenchymal Stem Cells: A Systematic Review of Safety and Efficacy. J. Funct. Biomater..

[2] Blaga F.N., Nutiu A.S., Lupsa A.O., Ghiurau N.A., Vlad S.V., Ghitea T.C. (2024). Exploring Platelet-Rich Plasma Therapy for Knee Osteoarthritis: An In-Depth Analysis. J. Funct. Biomater..

[3] Weiss M., Schroeder S., Zierke J., Damm P., Kretzer J.P. (2025). Lightening Their Load: Considering Overweight Complications in Patients with Hip Implants. Expert Rev. Med. Devices.

[4] World Health Organization (2020). Decade of Healthy Ageing: The Global Strategy and Action Plan on Ageing and Health 2016–2020.

[5] Organisation for Economic Co-operation and Development (OECD) (2021). Health at a Glance 2021: OECD Indicators.

[6] Svedbom A., Hernlund E., Ivergård M., Compston J., Cooper C., Stenmark J., McCloskey E.V., Jönsson B., Kanis J.A. (2013). Osteoporosis in the European Union: A Compendium of Country-Specific Reports. Arch. Osteoporos..

[7] Royal College of Physicians National Hip Fracture Database Annual Report 2019. https://www.nhfd.co.uk/files/2019ReportFiles/NHFD_2019_Annual_Report_v101.pdf.

[8] Learmonth I.D., Young C., Rorabeck C. (2007). The Operation of the Century: Total Hip Replacement. Lancet.

[9] Popov V.L., Poliakov A.M., Pakhaliuk V.I. (2023). Is It Possible to Create an “Ideal Endoprosthesis” for an “Ideal Total Hip Replacement”?. Prosthesis.

[10] Erickson A.K., Orona C.E., Parker E.M., Dyce J. (2025). Factors Affecting the Candidacy for Total Hip Replacement Assessed in 953 Dogs. VCOT Open.

[11] Panfilov I., Vilkovyskiy I., Sadyrin E., Aizikovich S., Beskopylny A.N., Meskhi B. (2025). Stress–Strain State Investigation and Ultimate Load on Femoral Implants Based on S-Type Ti6Al4V Titanium Alloy. J. Funct. Biomater..

[12] Tidwell S.J., Barnett R.J., Marcellin-Little D.J., Peck J.N. (2025). Accuracy of Dog Positioning before Total Hip Replacement Is High and Not Influenced by Surgical Experience. Am. J. Vet. Res..

[13] Inoue Y., Kuroda K. (2025). Successful Reimplantation of a Femoral Stem Fracture after Cementless Total Hip Replacement Using the Femoral Window Technique in a Small Dog. Animals.

[14] Ginja M., Gaspar A.R., Ginja C. (2015). Emerging Insights into the Genetic Basis of Canine Hip Dysplasia. Vet. Med. Res. Rep..

[15] Rettenmaier J.L., Keller G.G., Lattimer J.C., Corley E.A., Ellersieck M.R. (2002). Prevalence of Canine Hip Dysplasia in a Veterinary Teaching Hospital Population. Vet. Radiol. Ultrasound.

[16] Loder R.T., Todhunter R.J. (2017). The Demographics of Canine Hip Dysplasia in the United States and Canada. J. Vet. Med..

[17] Ginja M.M.D., Silvestre A.M., Colaço J., Gonzalo-Orden J.M., Melo-Pinto P., Orden M.A., Ferreira A.J. (2009). Hip Dysplasia in Estrela Mountain Dogs: Prevalence and Genetic Trends 1991–2005. Vet. J..

[18] (2025). Coherent Market Insights. Veterinary Orthopedics Market Size and Share Analysis—Growth Trends and Forecasts (2025–2032). https://www.coherentmarketinsights.com/industry-reports/veterinary-orthopedics-market.

[19] Grand View Research (2024). Europe Veterinary Orthopedic Medicine Market Outlook, 2030. https://www.grandviewresearch.com/horizon/outlook/veterinary-orthopedic-medicine-market/europe.

[20] Skurla C.P., Pluhar G.E., Frankel D.J., Egger E.L., James S.P. (2005). Assessing the Dog as a Model for Human Total Hip Replacement: Analysis of 38 Canine Cemented Femoral Components Retrieved at Post-Mortem. J. Bone Jt. Surg. Br..

[21] Bloebaum R.D., Ota D.T., Skedros J.G., Mantas J.P. (1993). Comparison of Human and Canine External Femoral Morphologies in the Context of Total Hip Replacement. J. Biomed. Mater. Res..

[22] Varut R.M., Trasca D.M., Stoica G.A., Sirbulet C., Arsenie C.C., Popescu C. (2025). Animal Models as Foundational Tools in Preclinical Orthopedic Implant Research. Biomedicines.

[23] Pearce A.I., Richards R.G., Milz S., Schneider E., Pearce S.G. (2007). Animal Models for Implant Biomaterial Research in Bone: A Review. Eur. Cells Mater..

[24] Powers D.L., Claassen B., Black J. (1995). The Rat as an Animal Model for Total Hip Replacement Arthroplasty. J. Investig. Surg..

[25] Ferguson R.J., Palmer A.J., Taylor A., Porter M.L., Malchau H., Glyn-Jones S. (2018). Hip Replacement. Lancet.

[26] Merola M., Affatato S. (2019). Materials for Hip Prostheses: A Review of Wear and Loading Considerations. Materials.

[27] Affatato S., Spinelli M., Squarzoni S., Traina F., Toni A. (2009). Mixing and Matching in Ceramic-on-Metal Hip Arthroplasty: An In Vitro Hip Simulator Study. J. Biomech..

[28] Taylor S.K. (1999). In Vitro Wear Performance of a Contemporary Alumina–Alumina Bearing Couple under Anatomically Relevant Hip Joint Simulation. Reliability and Long-Term Results of Ceramics in Orthopedics.

[29] Shetty V., Shitole B., Shetty G., Thakur H., Bhandari M. (2011). Optimal Bearing Surfaces for Total Hip Replacement in the Young Patient: A Meta-Analysis. Int. Orthop..

[30] McCarroll T.R., Kuhns B.D., Domb B.G. (2025). Surgical Management of Hip Pain in Active Patients with Early Osteoarthritis. JAAOS-J. Am. Acad. Orthop. Surg..

[31] Koh Y.G., Lee J.A., Kang K.T. (2019). Prediction of Wear on Tibial Inserts Using Finite-Element Analysis. Lubricants.

[32] Katti K.S. (2004). Biomaterials in Total Joint Replacement. Colloids Surf. B.

[33] Kurtz S.M. (2004). The UHMWPE Handbook.

[34] Laska A., Archodoulaki V.M., Duscher B. (2016). Failure Analysis of Retrieved PE-UHMW Acetabular Liners. J. Mech. Behav. Biomed. Mater..

[35] Shahemi N., Liza S., Abbas A.A., Merican A.M. (2018). Long-Term Wear Failure Analysis of UHMWPE Acetabular Cup. J. Mech. Behav. Biomed. Mater..

[36] Stratton-Powell A.A., Pasko K.M., Brockett C.L., Tipper J.L. (2016). Biologic Response to PEEK Wear Particles: A Review. Clin. Orthop. Relat. Res..

[37] Vaishya R., Vaish A., Dubey A., Vishwanathan K., Haleem A., Javaid M., Migliorini F. (2026). Role of PEEK in Arthroplasty and Orthopaedics. Eur. J. Orthop. Surg. Traumatol..

[38] Dallal S., Eslami B., Tiari S. (2025). Advances in PEEK for Biomedical Applications. Polymers.

[39] Kilina P., Kuchumov A.G., Sirotenko L., Vassilouk V., Golovin S., Drozdov A., Sadyrin E.V. (2024). Influence of Porous Titanium-Based Jaw Implant Structure on Osseointegration. J. Mech. Behav. Biomed. Mater..

[40] Aherwar A., Singh A.K., Patnaik A. (2016). Cobalt-Based Alloy as Biomaterial for Hip Implants. Trends Biomater. Artif. Organs.

[41] Manthe J., Cheng K.Y., Bijukumar D., Barba M., Pourzal R., Neto M., Mathew M.T. (2022). CoCrMo Alloy Microstructure in Hip Implants. J. Mech. Behav. Biomed. Mater..

[42] Fellah M., Labaïz M., Assala O., Dekhil L., Taleb A., Rezag H., Iost A. (2014). Tribological Behavior of Ti-6Al-4V and Ti-6Al-7Nb Alloys for Total Hip Prosthesis. Adv. Tribol..

[43] Şensoy A.T., Çolak M., Kaymaz I., Findik F. (2019). Optimal Material Selection for Hip Implant. Arab. J. Sci. Eng..

[44] Levine B., Della Valle C.J., Jacobs J.J. (2006). Applications of Porous Tantalum. JAAOS-J. Am. Acad. Orthop. Surg..

[45] Bobyn J.D., Toh K.K., Hacking S.A., Tanzer M., Krygier J.J. (1999). Tissue Response to Porous Tantalum. J. Arthroplast..

[46] Esfahani A.H., Akouchakian E., Sayyedan F.S. (2026). Ceramic Coatings for Implants Used in Total Hip Replacement: A Review on Materials and Methods. Biomed. Mater. Devices.

[47] Di Carlo G., Gorgone M., Salinari M., Minerba A., Castagnini F., Traina F. (2026). Revision Hip Arthroplasty after Fractured Ceramic Bearings Using Ceramic-On-Ceramic Surfaces: Long-Term Clinical and Radiological Outcomes of 36 cases. J. Arthroplast..

[48] Prasad K.N., Ramkumar P. (2023). FEM Wear Prediction of Ceramic Hip Bearings. J. Mech. Behav. Biomed. Mater..

[49] Forental G.A., Sapozhnikov S.B. (2024). Increasing the Interlayer Fracture Toughness of Polymer Fabric Composites Using Local 3D-Reinforcement (Felting). Adv. Eng. Res..

[50] Lee G. (2017). Ceramic Component Fracture. Orthop. Proc..

[51] Fernández-Fairén M., Torres-Perez A., Perez R., Punset M., Molmeneu M., Ortiz-Hernández M., Gil J. (2020). Mechanical Failures of Ceramic Bearings. Materials.

[52] Bader R., Willmann G. (1999). Ceramic Cups for Hip Endoprostheses. Biomed. Technol..

[53] Affatato S., Traina F., Mazzega-Fabbro C., Sergo V., Viceconti M. (2009). Ceramic-on-Ceramic Squeaking Phenomenon. J. Biomed. Mater. Res. B.

[54] Piconi C., Maccauro G. (1999). Zirconia as a Biomaterial. Biomaterials.

[55] De Aza A.H., Chevalier J., Fantozzi G., Schehl M., Torrecillas R. (2002). Crack Growth Resistance of Ceramics. Biomaterials.

[56] V@Art Cementless Hip Replacement System for Animals. https://v-art.info/.

[57] Alipour S., Moridi A., Liou F., Emdadi A. (2022). Additively Manufactured Titanium Alloys. Addit. Manuf..

[58] Shunmugavel M., Polishetty A., Goldberg M., Singh R., Littlefair G. (2017). Mechanical Properties of Ti-6Al-4V. Rapid Prototyp. J..

[59] Kurpiel S., Zagórski K., Cieślik J., Skrzypkowski K., Kapayeva S., Torekhanova M. (2023). Dimensional Deviations of Ti Alloy. Materials.

[60] Sadyrin E.V., Nikolaev A.L., Chapek S.V., Nazarenko D.V., Aizikovich S.M., Wang Y.C. (2023). Manufacturing Quality Evaluation of 3D-Printed Scaffolds. Generalized Continua.

[61] Jeong T., Shin H.J. (2014). An approximation technique for real-time rendering of Phong reflection model with image-based lighting. J. Korea Comput. Graph. Soc..

[62] Oliver W.C., Pharr G.M. (1992). An Improved Technique for Determining Hardness. J. Mater. Res..

[63] Oken O.F., Soydan Z., Yildirim A.O., Gulcek M., Ozlu K., Ucaner A. (2011). Performance of Modified Plates. Injury.

[64] Griffin B.J. (2011). Comparison of SEM Detectors. Scanning.

[65] Kværndrup F.B., Engelbrekt C., Kücükyildiz Ö.C., Somers M.A., Christiansen T.L., Winther G. (2022). Nanoindentation of Titanium. Mater. Today Commun..

[66] Singh S.P., Smith J.F., Singh R.P. (2008). Damping Behavior of Nanoindentation Instrument. Exp. Mech..

[67] (1991). Titanium and Titanium Alloys, Wrought. Grades.

[68] Ortiz-Marqués A., Caldevilla P., Goldmann E., Safuta M., Fernández-Raga M., Górski M. (2025). Porosity and permeability in construction materials as key parameters for their durability and performance: A review. Buildings.

[69] Marzec M., Duda P., Wróbel Z. (2020). Analysis of microtomographic images in automatic defect localization and detection. Mach. Vis. Appl..

[70] Chauhan S., Rühaak W., Khan F., Enzmann F., Mielke P., Kersten M., Sass I. (2016). Processing of rock core microtomography images: Using seven different machine learning algorithms. Comput. Geosci..

[71] Swain M.V., Xue J. (2009). Micro-CT Applications in Dental Research. Int. J. Oral Sci..

[72] Qin L., Genant H.K., Griffith J.F., Leung K.S. (2007). Advanced Bioimaging Technologies.

[73] Zelentsov V.B., Sadyrin E.V., Mitrin B.I., Swain M.V. (2023). Mathematical Tools Based on Micro-CT. J. Mech. Behav. Biomed. Mater..

[74] Khosravani M.R., Reinicke T. (2020). X-ray CT in 3D-Printed Components. J. Nondestruct. Eval..

[75] Nouri H., Guessasma S., Belhabib S. (2016). Structural Imperfections in AM. J. Mater. Process. Technol..

[76] Elenskaya N., Vindokurov I., Sadyrin E., Nikolaev A., Tashkinov M. (2024). Polymer Scaffold Degradation. Polymers.

[77] Niinomi M., Liu Y., Nakai M., Liu H., Li H. (2016). Biomedical Titanium Alloys. Regen. Biomater..

[78] Trofimov E.A., Lutfullin R.Y., Kashaev R.M. (2015). Elastic Properties of Ti-6Al-4V. Lett. Mater..

[79] Leyens C., Peters M. (2003). Titanium and Titanium Alloys: Fundamentals and Applications.

[80] Lee Y.T., Peters M., Welsch G. (1991). Elastic moduli and tensile and physical properties of heat-treated and quenched powder metallurgical Ti-6Al-4V alloy. Metall. Trans. A.

[81] Kasperovich G., Hausmann J. (2015). Improvement of fatigue resistance and ductility of TiAl6V4 processed by selective laser melting. J. Mater. Process. Technol..

[82] Guler M.A., Erdogan F. (2004). Contact mechanics of graded coatings. Int. J. Solids Struct..

[83] Schwarzer N. (2006). The extended Hertzian theory and its uses in analyzing indentation experiments. Philos. Mag..

[84] Krenev L.I., Volkov S.S., Sadyrin E.V., Zubar’ T.I., Chizhik S.A. (2018). Mechanical Material Tests by the Nanoindentation Method at Various Indenter and Specimen Temperatures. J. Eng. Phys. Thermophys..

[85] Vatul’yan A.O., Kossovich E.L., Plotnikov D.K. (2017). Some specific characteristics of indentation of cracked layered structures. Mech. Solids.

[86] Vasiliev A.S., Volkov S.S., Belov A.A., Litvinchuk S.Y., Aizikovich S.M. (2017). Indentation of a hard transversely isotropic functionally graded coating by a conical indenter. Int. J. Eng. Sci..

[87] Chudoba T., Jennett N.M. (2008). Higher accuracy analysis of instrumented indentation data obtained with pointed indenters. J. Phys. D Appl. Phys..

[88] Lewis G.S., Mischler D., Wee H., Reid J.S., Varga P. (2021). Finite Element Analysis of Fracture Fixation. Curr. Osteoporos. Rep..

[89] Wehner T., Claes L., Niemeyer F., Nolte D., Simon U. (2010). Influence of Fixation Stability on Healing. Clin. Biomech..

[90] Li J., Zhao Z., Yin P., Zhang L., Tang P. (2019). Internal Fixation Implants Comparison. J. Orthop. Surg. Res..

[91] Liao Y., Pourzal R., Stemmer P., Wimmer M.A., Jacobs J.J., Fischer A., Marks L.D. (2012). Hard Phases of CoCrMo. J. Mech. Behav. Biomed. Mater..

[92] Lone S.A., Xu D., Cook R.B., Hassel A.W. (2022). Cobalt-Based Alloys Properties. Phys. Status Solidi A.

[93] Yang J. (2025). Linear Theory for Small Deformation. A Concise Course in Elasticity.

[94] Ciarlet P., Luneville E. (2023). The Finite Element Method: From Theory to Practice.

[95] Tsybin N.Y. (2025). Exact and Approximate Stiffness Matrix and Nodal Load Vector for a Beam Finite Element with Linearly Varying Stiffness along Its Length. Adv. Eng. Res..

[96] Gere J.M. (2004). Mechanics of Materials.

[97] Scerrato D., Bersani A., Giorgio I., Allena R. (2026). A quasi-brittle strain-driven microdamage-informed remodelling approach for predicting proximal human femur damage and remodelling patterns. Math. Mech. Complex Syst..

[98] Lekszycki T., dell’Isola F. (2012). A mixture model with evolving mass densities for describing synthesis and resorption phenomena in bones reconstructed with bio-resorbable materials. ZAMM-J. Appl. Math. Mech./Z. Angew. Math. Mech..

